# Genome-Wide Profiling of *Cardinium*-Responsive MicroRNAs in the Exotic Whitefly, *Bemisia tabaci* (Gennadius) Biotype Q

**DOI:** 10.3389/fphys.2018.01580

**Published:** 2018-11-12

**Authors:** Hongran Li, Xiaoying Wei, Tianbo Ding, Dong Chu

**Affiliations:** Key Lab of Integrated Crop Pest Management of Shandong Province, College of Plant Health and Medicine, Qingdao Agricultural University, Qingdao, China

**Keywords:** *Bemisia tabaci* biotype Q, *Cardinium*, insect-symbiont interaction, microRNA, expression analysis

## Abstract

Although the bacterial symbiont *Cardinium* has profound effects on the ecological adaptation of its host, the whitefly *Bemisia tabaci* (Gennadius) biotype Q (hereafter referred to as *B. tabaci* Q), the molecular mechanism underlying interactions between these two organisms is not yet fully understood. In this study, sRNA libraries were constructed, amplified, and sequenced for *Cardinium*-infected (C^+^) and uninfected (C^∗−^) *B. tabaci* Q with identical genetic backgrounds. Subsequently, the genes targeted by the differentially expressed miRNAs were predicted by integrating the *B. tabaci* Q genome data. A total of 125 known and 100 novel miRNAs were identified, among which 23 significant differentially expressed miRNAs were identified in both libraries. It is noteworthy that an analysis of target genes showed that *Cardinium*-responsive miRNA-regulated genes were functional in apoptosis, reproduction, development, immune response, thermotolerance and insecticide resistance. GO and KEGG pathway analysis revealed that some miRNA-target genes are closely associated with energy metabolism. A major finding of this study was the identification of several miRNAs that may be involved in physiological processes in response to environmental stress, i.e., insecticides and high temperatures. This information will provide a foundation to help further elucidate the functions of *Cardinium* in *B. tabaci* Q.

## Introduction

Most phloem-feeding insects are infected with maternally inherited intracellular bacteria that have been categorized as either obligate symbionts or facultative symbionts (Gaelen et al., 2009). One of these facultative symbionts, *Candidatus Cardinium hertigii* (hereafter referred as *Cardinium*), was initially characterized in *Encarsia* wasps (Zchori-Fein et al., 2004). The symbiont has been shown to be a reproductive manipulator in several arthropod taxa through various means including cytoplasmic incompatibility (CI), feminization, and induction of parthenogenesis (White et al., 2011; Penz et al., 2012). Further, more recent research has demonstrated that *Cardinium* can also affect the fitness of its host (Harris et al., 2010; Fang et al., 2014).

Our long-term field surveys showed that the infection rate by *Cardinium* remains at a low level (12.2%) in the exotic sweet potato whitefly, *Bemisia tabaci* (Gennadius) Q (hereafter referred to as *B. tabaci* Q) in the Shandong Province, China (Chu et al., 2011). Further research revealed that competitive ability and fitness are diminished in *Cardinium*-infected *B. tabaci* populations compared to the *Cardinium*-uninfected population (Fang et al., 2014). Fitness (survival and reproductive success) of a field collected strain of *Cardinium*-infected *B. tabaci* Q, however, was shown to have unexpectedly improved after being reared under constant laboratory conditions for > 3 years (Li Hong-Ran, unpublished data). We speculated that the potential negative effect of *Cardinium* on its host whitefly may be the result of environmental stress under field conditions, i.e., in response to insecticides and high temperatures. To date, little information is available regarding the molecular mechanism underlying the *Cardinium*-mediated phenotypes of their whitefly hosts (Li et al., 2018). A more thorough understanding of this mechanism will help understand the ecological adaptation and population dynamics of this introduced pest.

MicroRNAs (abbreviated as miRNAs) are numerous non-coding small RNAs (sRNAs) (20–24 nt) involved in the regulation of gene expression post-transcriptionally, through repression of mRNA translation or degradation of mRNA in the cytoplasm (Bühler et al., 2006; Bartel, 2009; Iliopoulos et al., 2009). As a result of ongoing research, the significant roles of miRNAs in the manipulation of metabolism, development, and epigenetic inheritance are becoming better understood (Esau et al., 2006; Carlsbecker et al., 2010; Wang et al., 2018). Accumulating evidence has indicated that miRNAs are involved in regulating diverse biological processes such as apoptosis, hematopoiesis, and patterning of the nervous and immune systems in various groups of insects (Bushati and Cohen, 2007; Asgari, 2013; Xu et al., 2017). More recently, dramatic changes in the expression levels of miRNAs have also been documented in response to symbiont and host interactions. For instance, the microRNA, aae-miR-2940-5p, which is highly enriched in *Wolbachia*-infected mosquitoes, may be involved in mediating the regulation of pelo in *Aedes aegypti* (Asad et al., 2018). Also, *Wolbachia* is able to use host miRNAs to regulate host gene expression, and manipulation of the mosquito metalloprotease gene via aae-miR-2940 is crucial for efficient maintenance of the endosymbiont (Hussain et al., 2011). An analysis of target genes in the two-spotted spider mite, *Tetranychus urticae*, showed that *Wolbachia*-responsive miRNAs regulate genes associated with lysosome function, apoptosis, sphingolipid metabolism, and lipid transport in both sexes, as well as reproduction in females (Rong et al., 2014). Because miRNAs are involved in infections by bacterial symbionts, such as *Wolbachia*, we postulated that the host miRNAs might also be involved in *Cardinium*-host interactions. A test of this hypothesis would provide insights into *Cardinium*-mediated phenotypes in host whiteflies.

Based on our current knowledge, sufficient data are available to characterize the profiles of miRNAs in whiteflies. Our previous results showed different expression levels of miRNAs in *B. tabaci* B vs. Q (Guo et al., 2013). Furthermore, Wang et al. (2016) investigated the expression profiles of miRNAs in *B. tabaci* B in response to viral infection. Still, insufficient research has been directed at exploring the potential role of miRNAs in *B. tabaci* following symbiont infection. In this study, we first established a *Cardinium*-infected *B. tabaci* Q strain (abbreviated as C^+^) and an uninfected strain (abbreviated as C^∗−^) with identical genetic backgrounds. Deep sequencing of four sRNA libraries was then conducted for both C^+^ and C^∗−^. Subsequently, bioinformatics analyses were carried out to categorize the sRNAs, assess differential expression of known and novel miRNAs, predict their targets and perform their functional annotation. These data will provide novel insight into the mechanism of insect-symbiont interactions, especially for *Cardinium* in *B. tabaci* Q.

## Materials and Methods

### Whitefly Colony

**FIGURE 1:**
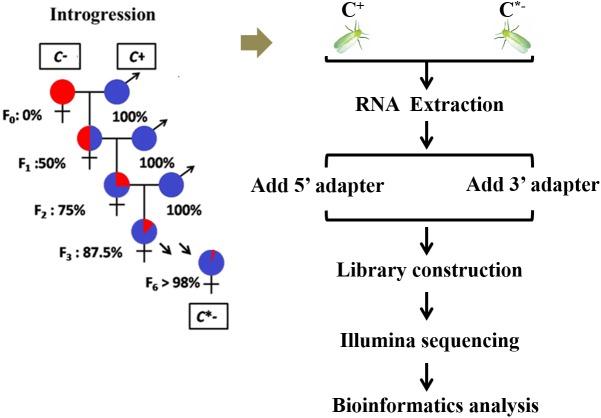
Experimental design and schematic diagram of the workflow used in this study.

The *B. tabaci* Q colony used in this study was established from individuals originally collected in the Shandong Province, China, in July 2012, and maintained in separate cultures for 3 years on potted cotton plants (Lu-Mian-Yan 21 cultivar). Whiteflies were kept in isolated screen cages under constant conditions of 27 ± 1°C, with a 16/8 h light/dark photoperiod, and 70 ± 5% RH. An introgressive backcrossing scheme was used to homogenize nuclear genetic backgrounds of infected and uninfected whiteflies for 6 generations, using the method described by Turelli and Hoffmann (1991). After completion of the introgression series, > 98% of nuclear alleles were expected to be shared between the C^+^ and C^∗−^ whiteflies; the two strains were then regarded as having a nearly identical genetic background (Figure 1). The one day-old adult females that were prepared for RNA isolation were selected from these *Cardinium*-infected and uninfected *B*. *tabaci* Q strains. To obtain newly emerged adult females, all adult whiteflies were removed from cotton leaves containing whitefly pupae each evening (at 6:00 pm). The following morning (at 8:00 am), newly emerged adults were individually collected and transferred into plastic tubes and sexed using a stereomicroscope (Luan et al., 2008; López and Andorno, 2009). All samples were stored at −80°C until needed for RNA isolation.

### *Cardinium* Detection in Populations

**FIGURE 2:**
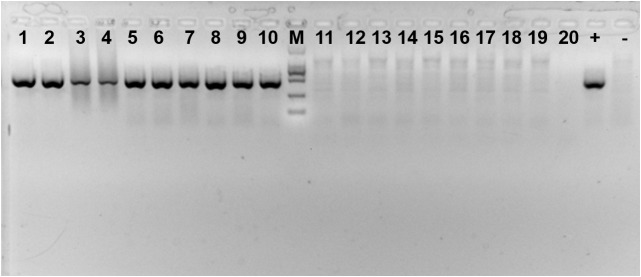
Amplification product of *Cardinium*-infected and uninfected *Bemisia tabaci* Q using specific primers. 1–10: *Cardinium*-infected *B. tabaci* Q; 11–20: *Cardinium*-uninfected *B. tabaci* Q; + : positive control; -: negative control; M: DL2000 DNA marker.

Presence of the symbiont *Cardinium* in each population was verified every 30 days from a sample of 20 adults using PCR primers to amplify a portion of the 16S rRNA gene (Figure 2). The primers used were CLO-F (5′- GCGGTGTAAAATGAGCGTG -3′) and CLO-R1 (5′- ACCTMTTCTTAACTCAAGCCT -3′) (Weeks et al., 2003). All PCRs were performed using 13 μL samples of a solution containing 1 × buffer, 0.16 mM of each dNTP, 0.5 mM of each primer, 0.5 unit Taq DNA polymerase (Takara, Dalian, China), and 2 μL template DNA. Cycling conditions consisted of initial denaturation at 95°C for five min, followed by 35 cycles of 1 min at 94°C for denaturation, 1 min at 58°C for annealing and 1 min at 72°C for elongation, plus a final extension step at 72°C for 7 min. All amplicons were electrophoresed, along with a negative (sterile water instead of DNA) and positive controls (DNA from previous sequencing) of the symbiont on a 1.0% agarose gel, and visualized using *Gelview* staining.

### Small RNA Library Construction for Illumina Sequencing

Total RNA was isolated from the C^+^ and C^∗−^ strains using the Trizol reagent (Invitrogen Catalog No. 15596-026) with a slight modification of the procedure recommended by the manufacturer. In this procedure, RNA was purified several times with isopropanol and 75% ethyl alcohol. Total RNA (>3 μg) of good quality was used to construct an sRNA library for each sample by using a TruSeq small RNA Sample Pre Kit (Illumina). Briefly, total RNA was ligated with 5′ and 3′ adapters followed by reverse transcription using RT primers. After PCR amplification of the cDNAs, amplified PCR products within the 130–160 bp size range were purified on an 8% polyacrylamide gel (100 V, 80 min). The libraries were constructed with purified RNAs and were sequenced on an Illumina HiSeq 2500/2000 platform at the Novogene Company, Beijing, China.

### Bioinformatics Analysis of Sequencing Data

After Illumina sequencing, raw data were processed using Novogene’ s Perl and Python scripts. Clean data were screened to remove reads containing more than three N (undetermined bases), reads with 5′ adapter contaminants, reads without 3′ adapter or the insert tags, those containing poly A, T, G, or C and low quality reads obtained from the raw data. Then, sRNA sequences of 18–35 nt were selected to conduct all downstream analyses. To prevent every unique sRNA mapping to multiple non-coding RNA (ncRNAs), we used the following priority rule: known miRNA > rRNA > tRNA > snRNA > snoRNA > repeat > gene > novel miRNA so that every unique sRNA mapped to only one annotation. The Bowtie software was used to map the sRNA tags to the *B. tabaci* Q genome^1^) with less than 2 bp mismatch to analyze their distribution and expression (Langmead et al., 2009).

Next, the mappable sRNA tags were used to search the release of version 20.0 of miRBase to identify known miRNAs in *B. tabaci*. Known miRNAs were defined as sequences that were identical to sequences from *Drosophila melanogaster* or other insects (*Aedes aegypti*, *Apis mellifera*, *Tribolium castaneum* and *Bombyx mori*) as outlined previously (Guo et al., 2013). Then, rRNAs, tRNAs, snRNAs, and snoRNAs were removed by mapping the remained sRNA tags to Rfam^2^. Repeat sequences were discarded by using a repeat sequence database^3^, and protein coding genes were filtered by mapping to the exon and intron of mRNAs of *B. tabaci*. Finally, novel miRNAs were predicted by exploring the secondary structure, the Dicer cleavage site and the minimum free energy of the former unannotated sRNA tags that could be mapped to the reference sequence by integrating two available software, miREvo and mirdeep2 (Wen et al., 2010; Friedlander et al., 2011).

### Differential Expression Analysis of miRNAs Between C ^+^ and C^∗−^ Strains

miRNA levels (transcripts per million; TPM) were estimated using the following normalization formula: Normalized expression = (mapped read count/total reads)^∗^1,000,000 (Zhou et al., 2010). Since there were two biological replicates for each sample, differential expression analysis of miRNAs between libraries was performed using the DESeq R package (1.8.3) (Anders and Huber, 2010). *P*-values were adjusted using the method of Benjamini and Hochberg (1995). The threshold level of collected *P*-values was set as 0.05 for significant differential expression.

### Target Prediction and Enrichment Analysis

We used the miRanda software to identify genes targeted by miRNAs in the genome of *B. tabaci*^4^ (Enright et al., 2003). To further reveal functions related to the putative target genes, GO and KEGG enrichment analysis of the predicted target genes was performed using the GOSeq/topGO2.12 and KOBAS 2.0 software (Mao et al., 2005; Kanehisa et al., 2008; Young et al., 2010).

The dataset that was studied in this article is available in the NCBI (SRA) public repository under accession number SRP076077.

### Validation of Differentially Expressed miRNAs via qRT-PCR

Total RNA was extracted from the C^+^ and C^∗−^ whiteflies (taken from the same treatment samples as used for library construction). Reverse transcription was performed by Mir-X^TM^ miRNA First-Strand Synthesis Kit (Takara catalog no. 638313) under the following conditions: 37°C for 1 h, 85°C for 5 min, and then held at 4°C. Levels of miRNA were assessed in four biological and two technical replicates of each sample, using SYBR Premix EX TaqTM II (Takara catalog no. RR820A). The cDNA was quantified in 20μL reactions, containing 7.2 μL ddH_2_O, 10 μL SYBR Advantage Premix (2X), 0.8 μL miRNA-specific Primer (10 uM), mRQ 3′ Primer (supplied with the Kit No. RR820A) and 2 μL template cDNA. The primers used in this study are provided in Supplementary Table S1. The reactions were incubated at 95°C for 10 s, followed by 40 cycles of 95°C for 5 s, 60°C for 20 s. A dissociation curve was obtained to ensure that only one product was amplified after the amplification phase. The 2^−ΔΔCt^ method for relative quantification of gene expression was used to determine the level of miRNA expression.

## Results and Discussion

### Overview of the Analysis of sRNA Libraries

**FIGURE 3:**
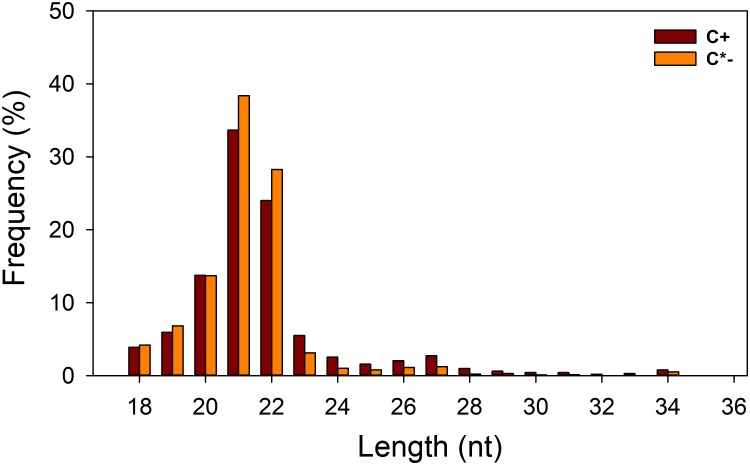
Length distribution of small RNAs in the two libraries. C ^+^, *Cardinium*-infected *B. tabaci* Q; C^∗−^, *Cardinium*-uninfected *B. tabaci* Q.

In this study, we generated two replicate sRNA libraries for each of the C^+^ and C^∗−^ strains (Table 1), totaling 44 million raw reads. About 43 million clean reads remained after the filtering step (see section “Materials and Methods” for details). Analysis of the length distribution among the 18–35 nt sRNAs examined here indicated that the highest proportion of small RNAs, in both strain-specific libraries, was in the 21–22 nt range (57.71 and 66.64% in the C^+^ and C^∗−^ libraries, respectively) (Figure 3). The proportion of small RNAs in the 21–22 nt range is consistent with that observed for miRNAs in animals (Li et al., 2011). But in previous studies examining miRNAs in *B. tabaci* B/Q and non-viruliferous whiteflies, length distribution showed maximum enrichment for 21–23 nt or 28–30 nt sRNAs (Guo et al., 2013; Wang et al., 2016). This difference may be due to sample treatment prior to library construction. Here, only one day-old adult females were utilized for total RNA isolation, whereas the whiteflies used in previous reports were neither isolated by sex nor segregated by time of emergence.

**Table 1:** Statistics of small RNA sequences in C^+^ and C^∗−^ libraries.

Group of reads	Number of sequences				Total
	C^+^1	C^+^2	C^∗−^1	C^∗−^2	
Raw data	11008300	11159082	10968649	11029471	44165502
Clean reads	10752028	10895385	10675963	10738383	43061759
Mapped total reads	9922549	10113477	10137941	10270736	40444703
Mapped unique reads	2610855	2500937	2786945	2825947	10724684
Mapped total sRNA	1234106	895124	1515005	1748357	5392592
Mapped unique sRNA	592	523	644	671	2430
Mapped hairpin	152	154	152	162	170
Mapped mature^§^	111	110	109	120	125
Novel miRNA	89	81	91	96	100
Novel miRNA^∗^	57	52	65	64	72

*^§^ indicates known miRNAs; ^∗^indicates passenger strand.*

**FIGURE 4:**
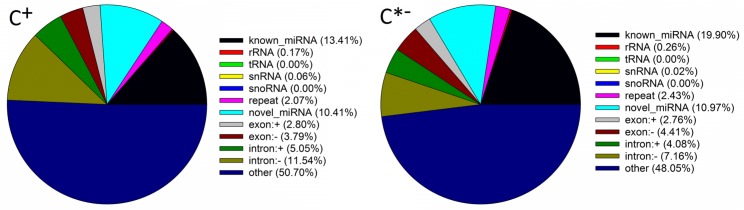
Classification of sRNAs in the C ^+^ and C^∗−^ libraries. snRNA, small nuclear RNA; snoRNA, small nucleolar RNA; exon: + , same orientation with mRNA exon; exon: -, reverse orientation with mRNA exon; intron: + , same orientation with mRNA intron; intron: -, reverse orientation with mRNA intron; other, unannotated sRNA.

The mapped sRNAs fell in one of 12 categories, including known miRNAs, rRNAs, tRNA, snRNA, snoRNA, repeat, novel miRNAs, exon, intron and unannotated sRNAs (Figure 4). The percentages of known miRNAs in the C^∗−^ libraries (19.90%) were higher than those in the C^+^ libraries (13.41%), whereas the corresponding values for novel miRNAs were similar (10.97 and 10.41%, respectively). Thus, differences of both length distribution and composition of two sRNA libraries suggest that infection by *Cardinium* may inhibit the synthesis of miRNAs in *B. tabaci*. The same results were found in *T. urticae* infected with the symbiont *Wolbachia* (Rong et al., 2014).

### Identification of Known and Novel miRNA

Sequences in our libraries identical to miRNA sequences of *D. melanogaster*, *Aedes aegypti*, *Apis mellifera*, *T. castaneum* and *B. mori* were considered to be potentially known miRNAs. Following a BLASTn search against the miRBase 20.0 and subsequent sequence analysis, a total of 125 known miRNAs were identified in the four libraries (Table 1 and Supplementary Table S2). Next, we used the miRNA prediction software miRDeep2 to identify putative novel miRNAs by searching against the *B. tabaci* Q genome sequence^5^. In total, 100 novel miRNAs were identified from both libraries (Table 1). To determine whether these novel miRNAs are known in other species, we used these miRNA sequences to search against all nucleotide sequences in miRBase Version 20.0. The results showed that 79 novel miRNA sequences of *B. tabaci* were similar to those in other species, but differed in some nucleotide positions, while 21 novel miRNA sequences were not found in other species (Supplementary Table S3). Thus, 21 novel miRNAs are putatively specific to whiteflies and could thus have a species-specific functions.

**FIGURE 5:**
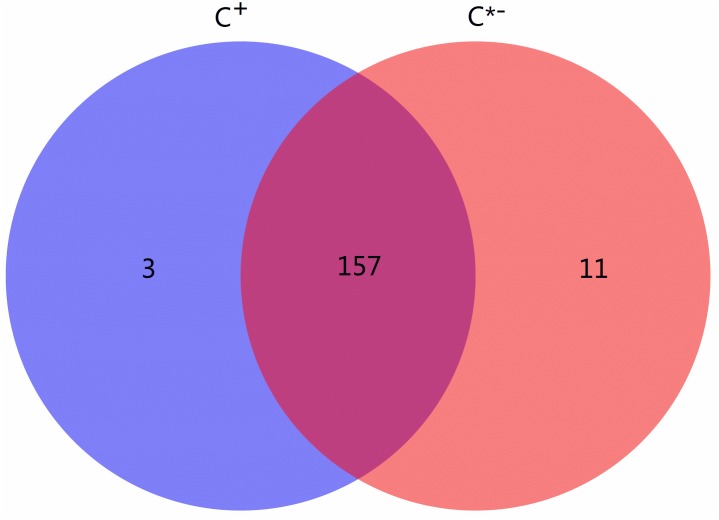
Venn chart of miRNA expression in the C ^+^ vs. C^∗−^ strains. The blue circle represents C ^+^, *Cardinium*-infected *B. tabaci* Q; the red circle represents C^∗−^, *Cardinium*-uninfected *B. tabaci* Q.

Among all identified known and novel miRNAs, three miRNAs [novel-122 (efu-miR-9216), novel-156 (hsa-miR-4762-3p) and tca-miR-263b-5p] were detected only in C^+^ and 11 miRNAs were found only in C^∗−^, while 157 miRNAs were found in both libraries (Figure 5). miR-263a/b affects expression of genes contributing to cellular and humoral immunity in *Manduca sexta* and regulates immunity-related signal transduction by affecting the expression of genes related to the *Galleria mellonella* tumor necrosis factor receptor superfamily, suggesting that the specific tca-miR-263b-5p in *Cardinium*-infected *B. tabaci* Q may be a regulator of immune processes (Mukherjee and Vilcinskas, 2014; Zhang et al., 2014). An earlier study also reported that miR-263a/b negatively regulates apoptosis, chaeta development and compound eye morphogenesis in *D. melanogaster*, suggesting that tca-miR-263b-5p may play a role in cell apoptosis in C^+^ strain (Hilgers et al., 2010).

### Differentially Expressed miRNAs Between C^+^ and C^∗−^ Strains

**FIGURE 6:**
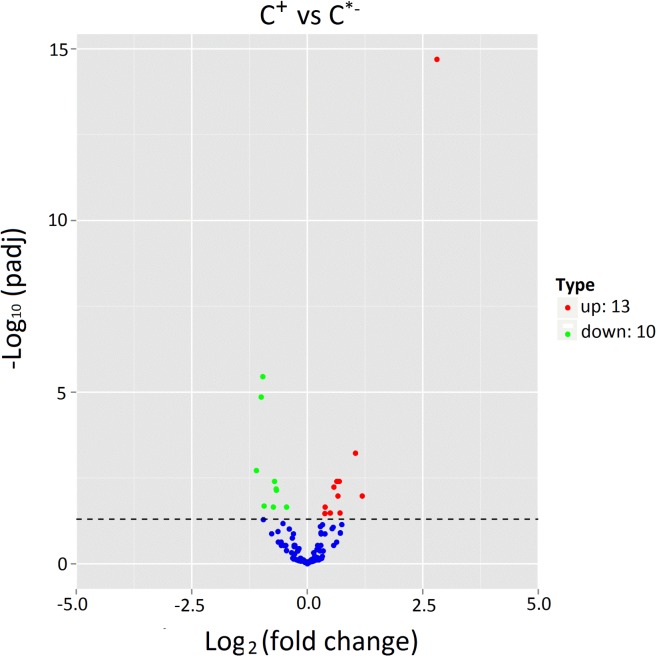
Expression of miRNAs in C^+^ vs. C^∗−^. The x-axis represents the fold change of miRNA expression in different samples; the y-axis represents the significance of the variances on transcription levels of miRNAs. Each point represents one miRNA. Red points represent the up-regulated miRNAs. Blue points indicate equally expressed miRNAs. Green points represent down-regulated miRNAs. *P* < 0.05.

The expression levels of known and novel miRNAs in C^+^ and C^∗−^ libraries were compared after normalization (normalized expression = actual miRNA count/total count of clean reads × 100000). We identified 23 differentially expressed miRNAs, including 13 up-regulated miRNAs and 10 down-regulated miRNAs in the C^+^ strain relative to the C^∗−^ strain (Table 2 and Figure 6). The strongest up-regulation was observed for novel-140, novel-27 (cte-miR-87b), novel-256 (ppc-miR-2235a-9-3p) and ame-bantam, while the strongest down-regulation was observed for novel-19, tca-miR-993-5p, bmo-miR-993b-5p and novel-193 (pma-miR-133a).

**Table 2:** List of all significantly differentially expressed miRNAs in C^+^ vs. C^∗−^ strains.

sRNA	Potential miRNA	Log2-fold change	Target gene id	Gene description
novel_140	^∗^	2.8072	BTA021458.1	Apoptosis-stimulating of p53 protein 2
			BTA017976.1	Glutathione S-transferase
			BTA011400.1	Heat shock protein 70 B2
novel_256	ppc-miR-2235a-9-3p	1.1904	BTA026695.3	Multidrug resistance-associated protein 4
			BTA029742.1	Cytochrome P450 9e2
			BTA019100.1	Activator of 90 kDa heat shock protein ATPase homolog 1
novel_27	cte-miR-87b	1.0441	BTA002500.1	Cytochrome P450 6k1
ame-bantam		0.70975		
novel_24	bmo-miR-13b-3p	0.69232	BTA004618.1	Multidrug resistance-associated protein 7
novel_1	ame-bantam	0.66426		
ame-miR-87		0.64842		
novel_152	pxy-miR-2b	0.63995		
novel_5	tca-miR-13a-3p	0.57691		
tca-miR-13a-3p		0.49866	BTA019714.1	Homeotic protein female sterile
ame-miR-281		0.38728		
aae-miR-277-3p		0.38109	BTA028304.1	Multidrug resistance protein MexA
dme-miR-277-3p		0.38109		
novel_19	^∗^	−1.1005	BTA013163.1	Non-lysosomal glucosylceramidase
			BTA027909.1	30S ribosomal protein S11
bmo-miR-993b-5p		−0.99651	BTA004944.1	Larval cuticle protein A1A
bmo-miR-993a-5p		−0.96172	BTA029040.1	Polyribonucleotide nucleotidyltransferase
tca-miR-993-5p		−0.96172	BTA017354.1	Pupal cuticle protein Edg-84A
novel_193	pma-miR-133a	−0.93463	BTA017001.1	Histone H3.3
novel_36	dps-miR-927-3p	−0.73363	BTA022535.1	Meiosis arrest female protein 1 homolog
novel_42	bmo-miR-2765	−0.70818	BTA027376.1	Apoptosis-resistant E3 ubiquitin protein ligase 1
dme-miR-305-5p		−0.67412	BTA013707.1	Adult-specific rigid cuticular protein 12.4
tca-miR-305-5p		−0.66718	BTA023288.2	Cuticle protein 8
novel_3	lva-miR-278-3p	−0.44769		

*Potential miRNA, miRNA sequences of B. tabaci that were similar to those in other species but differed in some nucleotide positions. ^∗^indicates miRNA sequences were not found in other species in miRBase Version 20.0.*

For up-regulated miRNAs, previous studies showed that bantam miRNA simultaneously stimulates cell proliferation and prevents apoptosis in *Drosophila*, indicating that an up-regulated level of ame-bantam in the C^+^ strain may help resist apoptotic cell death (Brennecke et al., 2003). miR-87 has been reported to be associated with anti-pathogen and immune responses (Liu et al., 2015). Thus up-regulation of novel-27 (cte-miR-87b) in the present study suggests it may play a role in immune responses in *Cardinium*-infected *B. tabaci* Q. With respect to down-regulated miRNAs, lmi-miR-133 was shown to mediate phenotypic plasticity and behavioral changes between social and solitary phases of henna and pale locusts by targeting key players in the dopamine synthesis pathway, indicating that novel-193 (pma-miR-133a) may modulate phenotypic plasticity in *B. tabaci* Q (Yang et al., 2014).

### Prediction of Target Genes for Differentially Expressed miRNAs

To further clarify the biological functions of the differentially expressed miRNAs, we analyzed the targets of 23 of them. A total of 12760 target genes for these 23 sRNAs listed in Table 2 were predicted. Among them, the number of target genes for each differentially expressed miRNAs ranged from 339 to more than one thousand. These results are consistent with previous findings indicating multiple miRNAs can be used to regulate a single gene and, conversely, that a single miRNA can target multiple genes, confirming the complexity of the miRNA-gene regulation network (Thomson et al., 2011).

**FIGURE 7:**
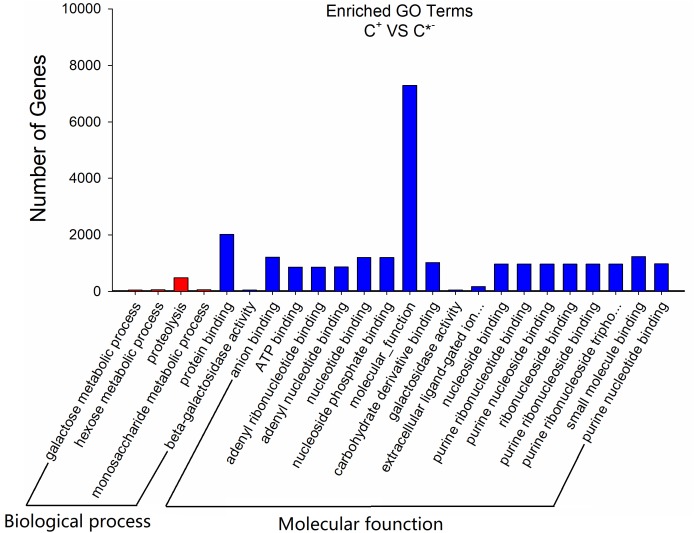
GO analysis results of the target genes of C^+^ vs. C^∗−^ strains. The *x*-axis is the GO category; the *y*-axis is the number of genes.

**FIGURE 8:**
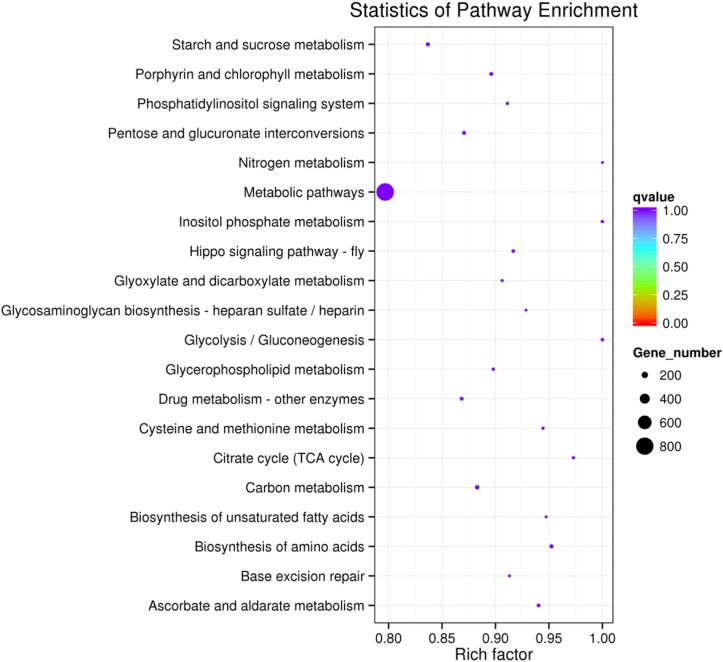
The 20 most enriched KEGG pathways based on target genes of differentially expressed miRNAs in C^+^ vs. C^∗−^ strains. The *x*-axis shows the rich factor. The *y*-axis shows the pathway names. The size of each point represents the number of genes enriched in a particular pathway. The larger the value of the rich factor and the smaller the value of *q*-value indicate a more significant degree of enrichment.

To further assess the presumptive functions of the genes predicted to be targeted by differentially expressed miRNAs, GO (Gene Ontology) annotation enrichment was performed. GO ontologies containing four different biological processes and 20 molecular functions were predicted (Figure 7 and Supplementary Table S4). The enrichment of each GO term within biological processes was compared, and most were involved in metabolic processes. As for molecular functions, the molecular function terms and most related binding terms comprised most of the targets, which is consistent with a regulatory role in transcription and translation for these miRNAs (Hobert, 2008). In addition, a KEGG (Kyoto Encyclopedia of Genes and Genomes) pathway analysis was preformed to elucidate the biological interpretation of the genes targeted by differentially expressed miRNAs (Figure 8). A total of 90 highly diversified biochemical pathways were identified that involved the miRNA-targeted genes, after considering a corrected *p*-value and the number of genes involved (Supplementary Table S5). Furthermore, a total of 1739 genes were enriched in the top 20 pathways. The “Metabolic pathways” category was the most significantly enriched term with 812 genes, followed by “Carbon metabolism” (98 genes) and “Starch and sucrose metabolism” (82 genes). The “rich factor” (x axis of Figure 8) represents the ratio of the number of differentially expressed genes to the total number of unigenes in the pathway. The most significant degree of enrichment was observed for the glycolysis “Glycolysis/Gluconeogenesis,” “Inositol phosphate metabolism” and “Nitrogen metabolism” pathways, all related to energy metabolism.

### *Cardinium*-Responsive miRNAs May Be Associated With Apoptosis Process in *B. tabaci* Q

The target genes of several significantly differentially expressed miRNAs in *B. tabaci* Q have been identified to be associated with apoptosis (Table 2). The gene BTA013163.1, targeted by down-regulated novel_19, encodes glucosylceramidase, whose deficiency can cause neurodegeneration and apoptosis in the brain of mice (Enquist et al., 2007). The observed down-regulation of novel_19 in C^+^ strain may indicate its role in inhibiting apoptosis. Moreover, the gene BTA021458.1 targeted by novel_140 codes for Apoptosis-stimulating of p53 protein 2. Therefore, the observed up-regulation of novel_140 in C^+^ strain may also indicate its role in decreasing apoptosis. It is well known that *Wolbachia* and *Cardinium* infection can lead to decreases in apoptosis in their respective hosts, *Drosophila mauritiana* and *Encarsia suzannae* (Fast et al., 2011; Mann et al., 2017). Female-biased genes encoding ribosomal proteins indicate an increase in general translational activity of *Cardinium* in female wasps (Mann et al., 2017). In this study, the gene BTA027909.1 targeted by down-regulated novel_19, encodes 30S ribosomal protein S11, indicating a potential increase in general translational activity of *Cardinium*. We, therefore, speculated that *Cardinium* in *B. tabaci* Q probably could inhibit apoptosis in its host, which in turn, would be beneficial to the maintenance of *Cardinium* itself.

### *Cardinium*-Responsive miRNAs May Regulate Reproduction and Development in *B. tabaci* Q

*Cardinium* can induce a number of reproductive disruptions in arthropods to optimize its transmission in a manner similar to that described for *Wolbachia*, including CI and feminization (Gotoh et al., 2007; White et al., 2011). CI was expected to an increase in the production of symbiont-infected females in the population (Poinsot et al., 2003; Werren et al., 2008). In the present study, some target genes of differentially expressed miRNAs were found to be related to female reproduction. For example, the target gene BTA017001.1 (targeted by novel_193) codes for Histone H3.3, which is believed to play an essential role in early developmental manifestations of CI (Serbus et al., 2008). In addition, the gene BTA022535.1, which is targeted by down-regulation of novel_36, codes for “meiosis arrest female protein 1 homolog” and is involved in oogenesis (Table 2).The miRNA tca-miR-13a-3p was up-regulated, which is expected to result in the down-regulation of BTA019714.1, a gene involved in female sterility. Moreover, target genes BTA013707.1 (targeted by dme-miR-305-5p), BTA017354.1 (targeted by tca-miR-993-5p), BTA023288.2 (targeted by tca-miR-305-5p) and BTA004944.1 (targeted by bmo-miR-993b-5p) code for cuticle protein in specific developmental stages. The down-regulated miRNAs should result in enhanced cuticular protein production, which may be related to growth and development. Thus, our results appear to support previous observations to the effect that *Cardinium* may manipulate host female reproduction and development, although this hypothesis will need to be further tested (Rong et al., 2014).

### *Cardinium*-Responsive miRNAs May Be Essential in *B. tabaci* Q Under Stress Conditions

Accumulating evidence indicates that miRNAs play an important role in drug resistance, as revealed by miRNA expression profiling (Xin et al., 2008; Ma et al., 2010; Zheng et al., 2010). In the present study, target gene BTA004618.1 (targeted by novel_24) codes for multidrug resistance-associated protein 7, and target genes such as BTA017976.1 (targeted by novel_140), BTA002500.1 (targeted by novel_27) and BTA029742.1 (targeted by novel_256) are related to glutathione S-transferase and cytochrome P450. In addition, target gene BTA026695.3 (targeted by novel_256) and BTA028304.1 (targeted by aae-miR-277-3p) are related to drug resistance as well. Those up-regulated miRNAs may reduce their targeted gene expression. Additionally, the target gene BTA011400.1 (targeted by novel_140) and BTA019100.1 (targeted by novel_256), encoding “Heat shock protein (HSP) 70 B2” and “Activator of 90 kDa HSP ATPase homolog 1.” The up-regulated miRNAs may result in reduced HSP protein production, which may depress tolerance. Taken together, our observations support our earlier speculation that the C^+^ strain may have lower thermotolerance, insecticide resistance and detoxification than the C^∗−^ strain.

### qPCR Validation of Differentially Expressed miRNAs

**FIGURE 9:**
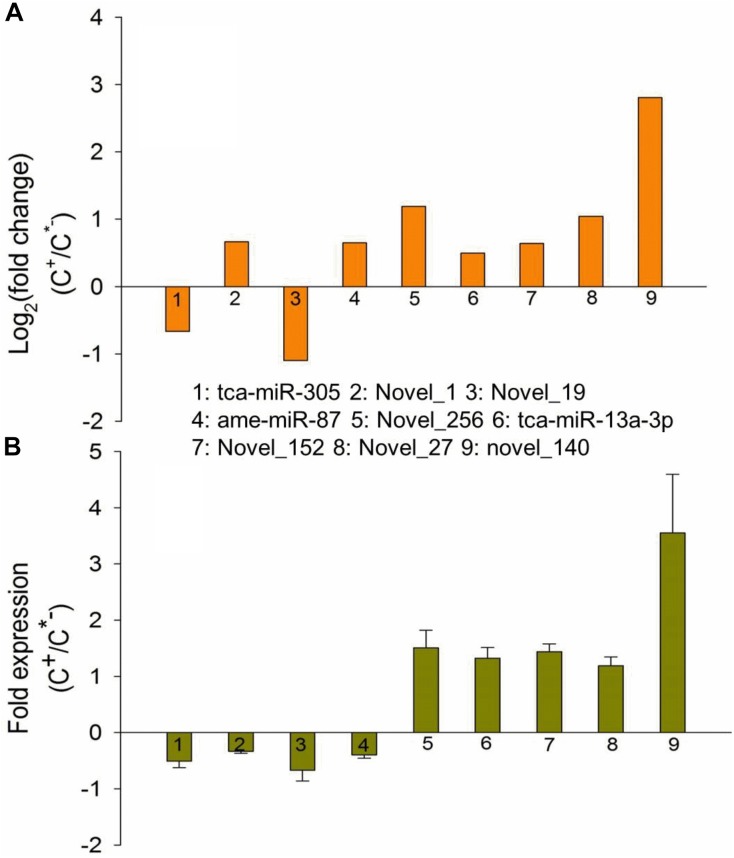
Expression patterns of differentially expressed miRNAs identified based on Illumina sequencing **(A)** and qRT-PCR **(B)**. *X*-axis, name of the miRNAs that were selected for qRT-PCR assessment; column above the *X*-axis: miRNAs that were up-regulated in the C^+^ strain; column below the *X*-axis: miRNAs that were upregulated in the C^∗−^ strain.

To further validate the expression patterns of miRNAs identified in this work, we randomly selected nine differentially expressed miRNAs to measure the expression levels via quantitative real time polymerase chain reaction (qRT-PCR). The results showed that seven miRNAs had qPCR expression patterns similar to those revealed by our RNA-seq analysis in the C^+^ and C^∗−^ strains. For example, novel-140 had been found to be highly expressed in the C^+^ strain, displaying a 2.8 log2-fold change compared with the C^∗−^ strain (Table 2 and Figure 9A), while similar results were obtained through a qPCR assessment, where approximately 4 times greater expression was found in the C^+^ strain (Figure 9B). However, two miRNAs, novel_1 and ame-miR-87, exhibited a decrease in expression in C^+^ as compared to C^∗−^ strain, which is the opposite of what the Illumina sequencing revealed. The reason for these differences is currently unknown. Overall, the qPCR results tend to indicate that the Illumina sequencing data accurately reflected the expression level of miRNAs.

## Conclusion

Using high-throughput sRNA sequencing, we screened out 23 miRNAs exhibiting differential expression in response to *Cardinium* infection in *B. tabaci* Q. These miRNAs are involved in several biological processes, including cell apoptosis, reproduction and development. The major finding of this study is the identification of several miRNAs overexpressed in infected whiteflies that target genes involved in thermotolerance and insecticide resistance, pointing to a compromised resistance to heat and xenobiotic stresses in C^+^ individuals. These findings may help explain fitness variation among *Cardinium*-infected *B. tabaci* Q whiteflies in response to environmental stress. In general, our findings lay a solid foundation for further functional study of the interactions between *Cardinium* and its host whitefly, *B. tabaci* Q.

## Ethics Statement

The present research complies with all laws of the country (China) in which it was performed and was approved by the Department of Science and Technology of the Qingdao Agricultural University, China (Permit Number: 20110712).

## Author Contributions

DC contributed to experimental design and management. HL carried out the data analysis and drafted the manuscript. XW and TD participated in data analysis. All authors read and approved the final manuscript.

## Conflict of Interest Statement

The authors declare that the research was conducted in the absence of any commercial or financial relationships that could be construed as a potential conflict of interest.

## Footnotes

1 http://gigadb.org/dataset/100286

2 http://rfam.xfam.org/

3 http://www.repeatmasker.org/cgi-bin/WEBRepeatMasker/

4 http://gigadb.org/dataset/100286

5 http://gigadb.org/dataset/100286
